# Coherent all-optical transistor based on frustrated total internal reflection

**DOI:** 10.1038/s41598-018-23367-6

**Published:** 2018-03-22

**Authors:** A. Goodarzi, M. Ghanaatshoar

**Affiliations:** Laser and Plasma Research Institute, Shahid Beheshti University, G.C., Evin, 1983969411 Tehran, Iran

## Abstract

This study aims to design an all-optical transistor based on tunneling of light through frustrated total internal reflection. Under total internal reflection, the electromagnetic wave penetrates into the lower index medium. If a medium with high refractive index is placed close to the boundary of the first one, a portion of light leaks into the second medium. The penetrated electromagnetic field distribution can be influenced by another coherent light in the low refractive index medium via interference, leading to light amplification. Upon this technique, we introduce coherent all-optical transistors based on photonic crystal structures. Subsequently, we inspect the shortest pulse which is amplified by the designed system and also its terahertz repetition rate. We will show that such a system can operate in a cascade form. Operating in terahertz range and the amplification efficiency of around 20 are of advantages of this system.

## Introduction

In recent years, interest in replacing conventional processors with photon-based ones has been increased. The reason is the reduce in processors’ speed growth rate and benefits of all-optical information transferring and processing^1–3^. The basic part of every integrated processor circuit is a transistor. Therefore, the first step is design and construction of an operative all-optical transistor. An all-optical transistor is a component in which an optical signal is amplified through an all-optical procedure^4,5^. Regarding the fact that photons do not interact with each other in vacuum, designing and manufacturing such devices will be a great challenge. In the design of an optical transistor, due to the importance of amplifying a signal and to supply the required energy, the system must be continually pumped^5^. In this case, the system is in standby mode although a portion of light leaks out of the system as output. When the gate signal is applied, it is expected to change the output of the system. For accomplishing the desired variation, the two beams should be different at least in one aspect, for example in intensity, wavelength, polarization and etc. Picking any of these light peculiarities will determine the transistor operation mechanism. On the other hand, some basic requirements should be fulfilled for an operational transistor to be usable in all-optical integrated systems. The important items include integrability, cascadability, working in low intensity, low loss, working in room temperature and etc.

Using nonlinear effects to design all-optical switches is one of the primary methods in this field^6–12^. However, high-intensity requirement and difficulty in achieving a gain value more than unit have reduced interests in manufacturing all-optical transistors through this intensity-based mechanism. Besides this kind of switches, several wavelength-based all-optical switches and transistors have also been designed so far^13–15^. Though some of them have many of the essential characteristics and can perform a high-level amplification, there is a problem with this approach which is the difference in input and output wavelengths. Thus, it is not possible to use the output of one transistor as the input of another similar one in a cascade configuration. Similarly, to have an integrable component on the basis of transistors working upon the polarization of light, polarization conversion after switching procedure is necessary for the circuit that complicates the system design. The technique based on the other two features (phase and direction) which are employed in this article, has not the mentioned disadvantages and can meet all the necessary conditions. However, since this method is based on the relative phase of the waves, coherency is an essential condition for pump and gate beams which makes necessary the use of a single light source. This kind of transistors is attractive in quantum computing and data transferring systems, so numerous attempts to provide practical transistors of this type have taken place in recent years^16–21^. In addition, some novel applications based on this method, like more accurate spectroscopy and localized absorption in metamaterials have been established^22–24^. When we benefit from the coherency of light, interference can help to achieve intensive interaction. Because of the square relation of the electric field amplitude and intensity of light, interference can lead to amplification in certain circumstances. In this method, the amplification magnitude (gain), response time and integrability are some of the important features that should be taken in mind. Despite the fact that some research has been carried out in this area, it is a challenge to meet these requirements, concurrently. For instance, up to now, the gain achieved by this technique was not enough to introduce an operative transistor^4,16,21,25–30^. To derive a transistor, the first one should have an efficiency more than 2^28^. Among the other complications, we can point to the free space configuration and non-integrable designs^4,25,29,31^.

In this article, we introduce a new approach to increase the influence of the signal on the pump beam. By using the frustrated total internal reflection (FTIR) and interaction of signal and the evanescent portion of the pump beam, we realize a significant gain. It is necessary to mention that the mechanism of work and physics governing the amplification procedure are essentially different from those introduced in one of our recent publications^5^. In that article, the phenomenon of coherent perfect absorption (CPA) was the dominant procedure by the presence of the chromium absorber layer, while there is no absorber layer in the present design and the fundamental mechanism for light amplification is the FTIR. We will show that besides acceptable response time and repetition rate, integrability is also accessible through this technique. In this order, we employ photonic crystal (PC) structures where light propagation within them is simulated using finite-difference-time-domain (FDTD) method.

## Theory

In interference of two optical beams, the resultant electric field is determined by vector summation rules^30,32^. Regarding square relation between the electric field and the light intensity, we can write:

1 $${I}_{p}={E}_{p}^{2}$$

2 $${I}_{g}={E}_{g}^{2}$$

3 $${I}_{tot}={({E}_{g}+{E}_{p})}^{2}={E}_{g}^{2}+{E}_{p}^{2}+2{E}_{g}{E}_{p}\,\cos \,\theta ={I}_{p}+{I}_{g}+2\sqrt{{I}_{p}{I}_{g}}\,\cos \,\theta $$

where *I*_*g*_ stands for the gate beam intensity, *I*_*p*_ is the pump light intensity and *I*_*tot*_ represents the system output in the presence of both gate and pump beams. When the gate and pump fields have the same phase in the desired location, the interference will be constructive, and so:

4 $${I}_{tot}={I}_{p}+{I}_{g}+2\sqrt{{I}_{p}{I}_{g}}$$

To examine the influence of the gate beam on the output signal, the variation in output by applying the gate beam is calculated. We examine this variation relative to the intensity of the gate pulse intensity as the gain of the system:

5 $${\rm{gain}}=\frac{{I}_{tot}-{I}_{p}}{{I}_{g}},$$

which equals to $1+2\sqrt{\frac{{I}_{p}}{{I}_{g}}}$ for instructive and $1-2\sqrt{\frac{{I}_{p}}{{I}_{g}}}$ for destructive interference of the beams. Therefore, depending on the relative amplitude and relative phase of the pump and gate beams at a given position, the change in the intensity of the output wave can be greater than the amplitude of the gate pulse.

**Figure 1:**
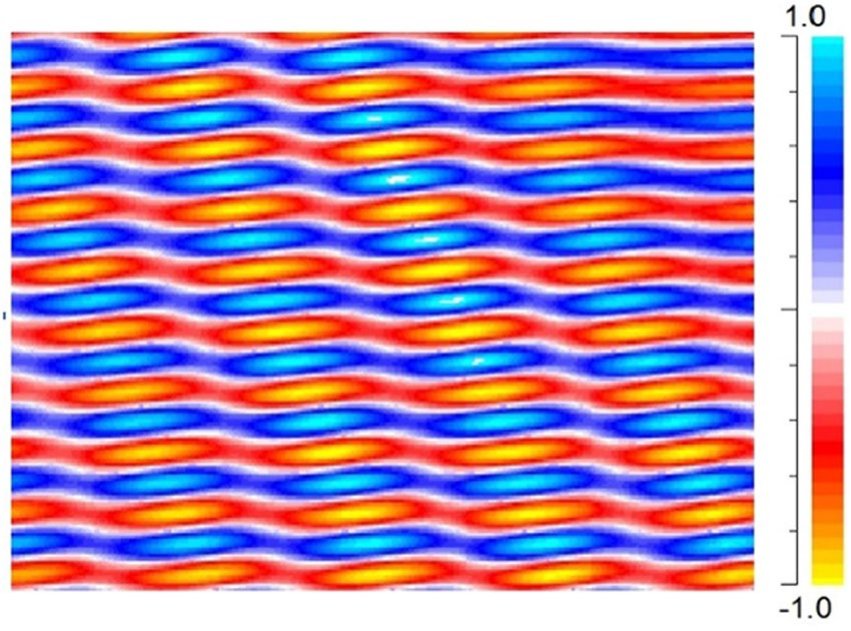
Free space interference of two wavefronts. The two waves are propagated with a relative propagation direction angle of 30° and 1 to 5 intensity ratio.

The interference pattern is determined by the wavefront of the waves and their propagation direction. To have a pure intensity variation by interference in free space, the wavefront and propagation direction of the interfering waves should be the same. Otherwise, destructive and constructive interferences occur periodically, which may disturb the exploitation of the interference pattern in a large space. Figure 1 shows interference of two waves with relative propagation direction angle of 30°, 1 to 5 intensity ratio, and planer wavefront.

**Figure 2:**
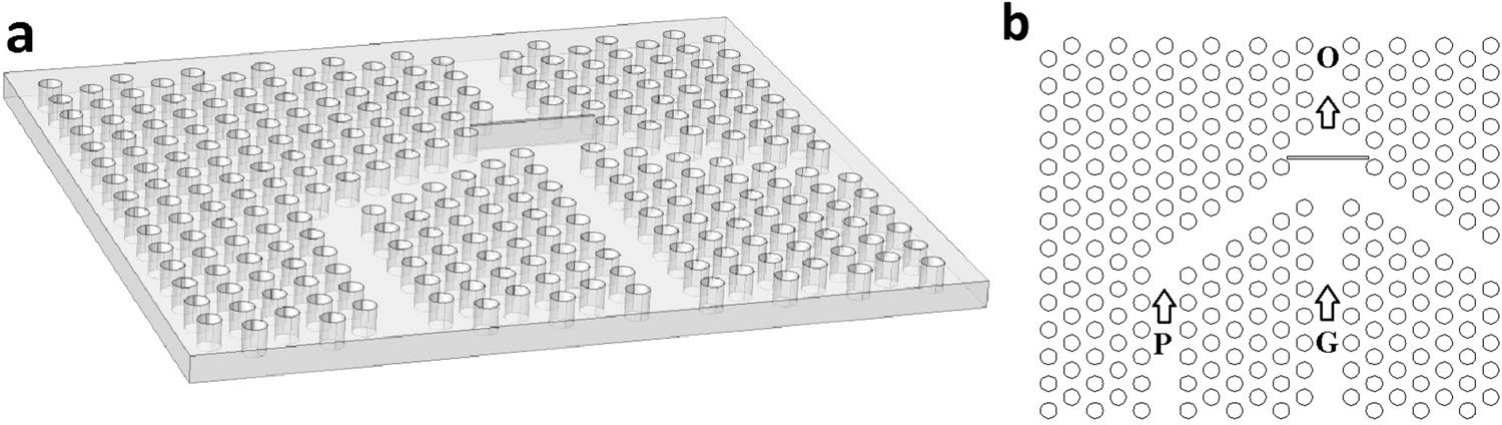
(**a**) The designed structure and (**b**) configuration of channels to conduct gate (G), pump (P) and output (O) beams.

Our solution to resolve this issue is using photonic crystal (PC) structures in order to limit the wavefront to a space with the size on the order of the wavelength^5,33^. For this purpose, a hexagonal air hole lattice in a silicon substrate is considered. As can be seen in Fig. 2, a very thin air gap is placed in the middle of the PC structure. We should explain that at present, manufacturing technologies with the required precision for creating such a thin air gap are available^23,34^. It is known that when a light wave strikes a medium boundary at an angle larger than a particularly critical one with respect to the normal to the surface, the total internal reflection occurs. If a high refractive medium is located at a small distance (on the order of the wavelength) from the surface, a portion of light penetrates into the medium which its intensity depends on the gap thickness. This tunneling of light occurs due to the presence of the evanescent waves at the air gap through FTIR mechanism. The intensity of the tunneled wave for an obliquely incident light can be determined by:

6 $${E}_{t}={E}_{0}{e}^{\mp \beta y}{e}^{i(\frac{{k}_{t}x{n}_{i}\sin {\theta }_{i}}{{n}_{t}}-\omega t)}$$

in which $\beta ={k}_{t}{(\frac{{n}_{i}^{2}{\sin }^{2}{\theta }_{i}}{{n}_{t}}-1)}^{\frac{1}{2}}$ and *θ*_*i*_ stands for the incident angle. By impinging a coherent gate beam at an angle smaller than the critical angle, we can change the penetration of light and in turn the evanescent electric field distribution in the air gap. The variation in the penetrated electric field distribution changes the intensity of the coherent pump beam in tunneling by interference. Therefore, according to the above equations, a properly designed PC structure can amplify the light intensity through tunneling and constructive interference.

### Simulation and results

**Figure 3:**
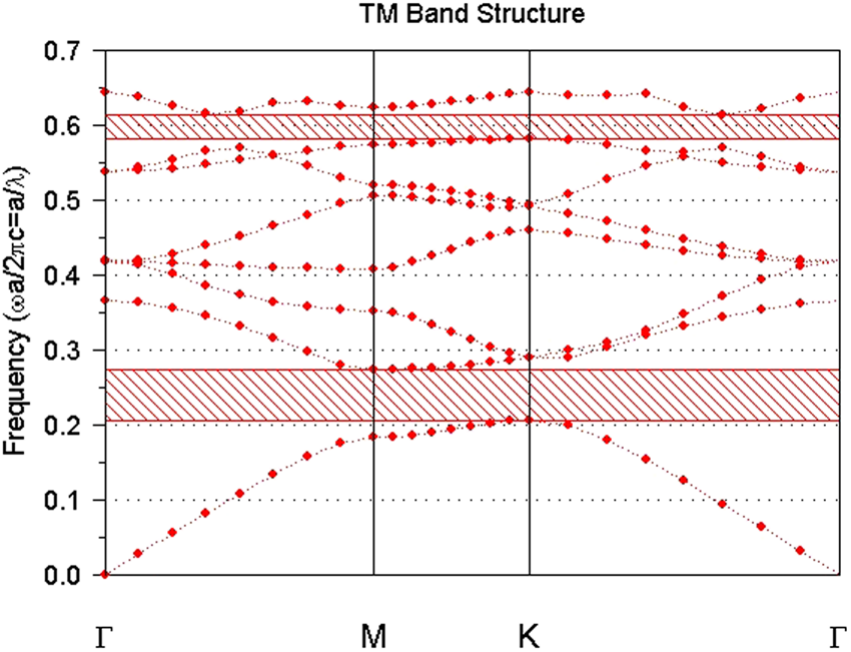
Photonic band structure of the exploited PC.

To simulate the propagation of light within the PC structure exhibited in Fig. 2, we employ the FDTD method. The working wavelength is considered to be 1550 nm and all simulations are performed in TM mode. The lattice constant “a” is 0.33 μm, the air hole radius is 0.3a and the refractive index of silicon at this wavelength is considered as 3.47. The photonic band structure of this silicon-based PC is illustrated in Fig. 3. This figure shows that the wavelength 1550 nm lies within the first band gap, which facilitates the propagation of this light through the channels.

**Figure 4:**
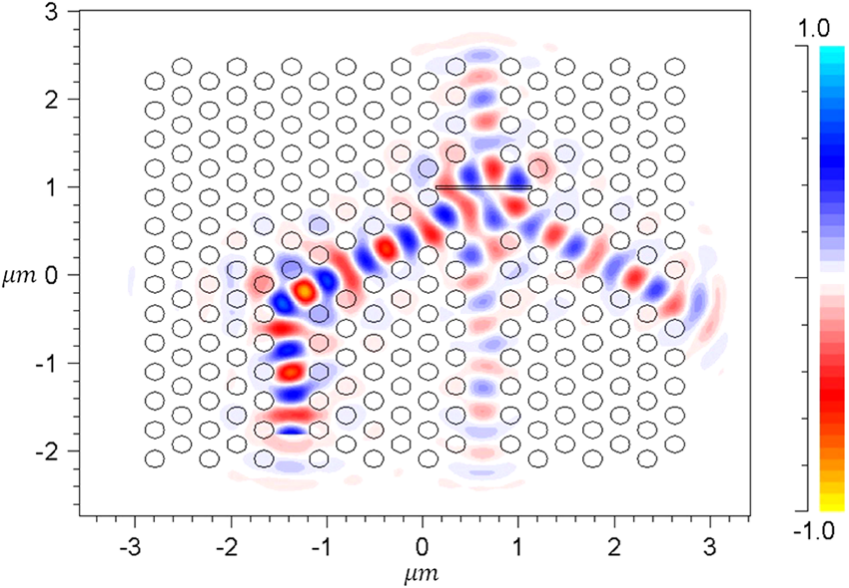
Propagation of pump beam within the PC structure.

In Fig. 2 we can see a rectangular air gap placed at the confluence of the gate and pump channels. It plays the role of a low refractive index layer which causes FTIR for the pump beam. This beam is propagated through P channel, indicated in Fig. 2, whereas the straight channel G transmits the gate beam. When a continuous pump wave is propagated lonely, a portion of this beam penetrates the output channel (O) through tunneling and scattering, and the remaining part is reflected and leaves the structure through the other channels. This situation provides a standby mode, which is seen in Fig. 4. In this case, impinging a gate beam on the air gap through G channel changes the field distribution within the PC. This beam can be a single pulse or a small signal on a continuous wave (CW) background. In simulations, we consider a unit amplitude CW pump beam whereas the amplitude of the gate pulse is 0.01 of the pump intensity. Moreover, the gate and pump waves are in phase at the position of the front boundary of the air gap. It should be noted that in the FDTD simulation of light propagation within the PC structure, demonstrated in Fig. 2, the grid size is considered to be a/44 and the time step is 0.01 fs. This is the case of structures with routine air gap width of about 25 nm and no sub-pixel smoothing is applied. For very thin air gaps (namely smaller than 10 nm), the grid size is taken to be a/80 and the time step is reduced to 0.006 fs to achieve the required accuracy.

**Figure 5:**
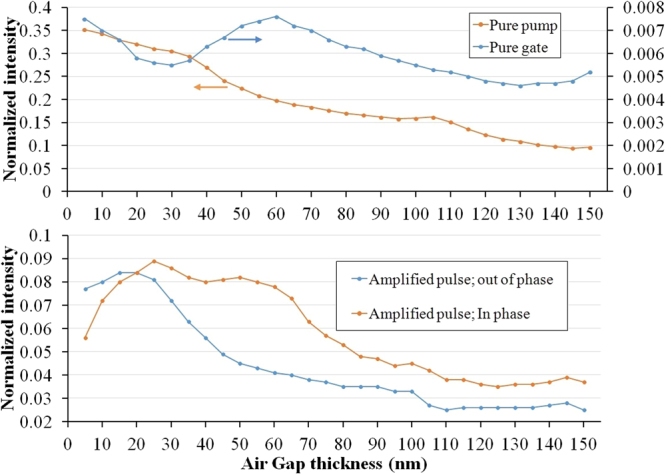
Output wave intensity (**a**) for pure pump and pure gate and (**b**) amplified pulse for in phase and out of phase waves at the first boundary of the air gap.

As our goal is to reach the maximum amplification of the gate pulse, firstly we look for the optimum air gap width by drawing the amplitude of the output pulse versus the air gap width. The change in the thickness of the air gap is made in such a way that the location of the first boundary of the air gap is fixed so that the variation of the electromagnetic field distribution at the front side of the gap will be minimal. Regarding the size of the system, we consider a 700 nm-thick slab for simulation. In comparison with a 2-dimensional structure, the output of such a thin slab exhibits a deviation less than 10% whereas, for small thicknesses, the leakage will deteriorate the performance of the device. For example, in the case of a 400 nm-thick slab, we will face a sharp drop in the intensity of the DC pump. Figure 5a displays the output wave intensity (normalized to unit power) at the output channel for the relative gate/pump input intensity of 0.01 whereas the input pump beam intensity is considered to be 1. Because of the collapse of the air gap within the PC lattice rods, it is not possible to increase the gap width beyond 150 nm. As it is observed, in the absence of the gate beam, the intensity of the transmitted pump wave decreases with some deviations from an exponential decrease which is expected from Eq. 6. This deviation is the result of light scattering from the rods and interference of the pump beam with itself in a photonic crystal structure. We should not forget that a portion of the passed light is the result of the scattered beam leakage. Regarding the FTIR mechanism, the transmission should be dropped for large air gaps in the absence of this leakage. As can be seen, the whole transmission does not go down to the zero because of this scattered radiation leakage. The situation for the pure gate is somehow similar, but from Fig. 2 it is clear that the gate beam propagates through the channel which is perpendicular to the air gap and obviously there is no internal reflection for the pure gate. In other words, the change in the gate transmission intensity is not related to the FTIR mechanism. Figure 5a demonstrates that in the absence of the pump beam, the portion of the gate pulse that enters the output channel decreases with the air gap thickness and reaches a minimum value. Although because of propagating in a structure and not free space we cannot find a harmonic behavior, the tale of the gate curve shows a tendency to come up.

The amplified pulse (presence of both gate and pump) for in phase and out of phase cases is plotted in Fig. 5b. As an important parameter, we have to give a value to the relative phase in every simulation. The relative phase of the two beams is considered to be constructive (in phase) at the first boundary of the air gap. In this figure, we also inspect the case of out of phase (destructive interference) for comparison. Since the phase is defined at the first boundary we did not expect to get an essentially different behavior versus the air gap width which is now deduced from this figure. By increasing the width of the thin air gap, the portion of pump beam that enters the output channel decreases exponentially. For small gaps, the dominant interaction is interference between the gate and pump beams and the tunneling phenomenon is negligible. Increase in the gap thickness makes the tunneling considerable and subsequently interaction of the gate and the evanescent pump waves becomes more significant. Since the tunneled wave amplitude varies exponentially with the thickness, a change in the pump beam electric field penetration due to interaction with the gate pulse causes an exponential alteration in the total transmitted intensity. Consequently, as shown in Fig. 5, despite the decline in the gate pulse amplitude entered into the output channel, the gain of the system is increased for the thickness range of 5–30 nm. This behavior is completely different from the gate intensity variation in the absence of the pump beam which reflects the governing of the FTIR mechanism. It should be noticed that in Eq. 3, *I*_*g*_ is the portion of the gate pulse that interacts with the pump beam in the air gap whereas the gain of the transistor is determined by the entered gate intensity. The penetrated light intensity decreases for thick air gaps and the gain will drop (see Fig. 5). The best value of the gain has been achieved for the gap thickness of around 25 nm. Its value for the case of in-phase beams is about 9 which is specific enough for transistor applications. Hereafter, in all simulations we consider a 25 nm-thick air gap.

**Figure 6:**
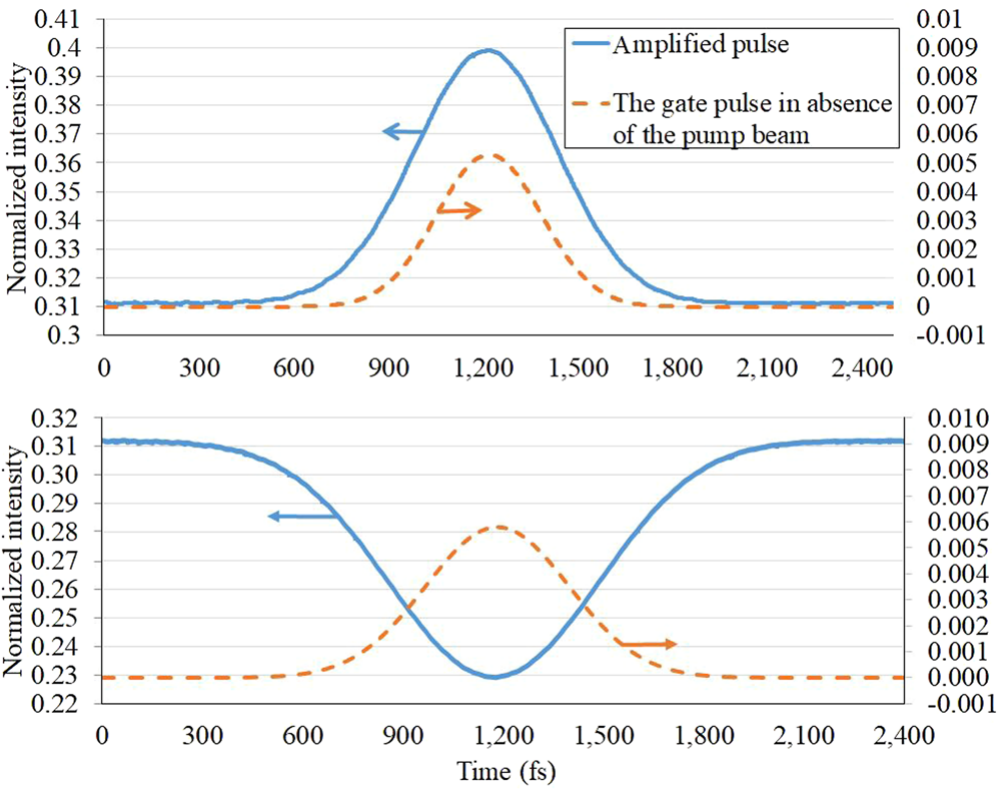
The gate pulse in output in the presence (amplified) and absence (pure gate) of the pump beam for (**a**) in phase and (**b**) out phase cases. The air gap is 25 nm-thick.

Despite the strength of the light amplification, the initial relative phase is determinant in the output pulse shape. Figure 6 illustrates the gate pulse in output in the presence of the pump beam (amplified) for in phase and out of phase cases. As can be deduced from this figure, the amplification does not lead to a pulse distortion for the in-phase case. Figure 6a demonstrates that the pulse shape is preserved during amplification process and its time delay is negligible. It just shows a slight broadening. Its full width at half maximum (FWHM) changes from 400 fs to 450 fs while the peak of the pulse grows up to 18 times. This quality of the output pulse is promising for making cascade transistors. On the other hand, Fig. 6b exhibits that the pulse shape declines from its original form by applying the out of phase condition.

**Figure 7:**
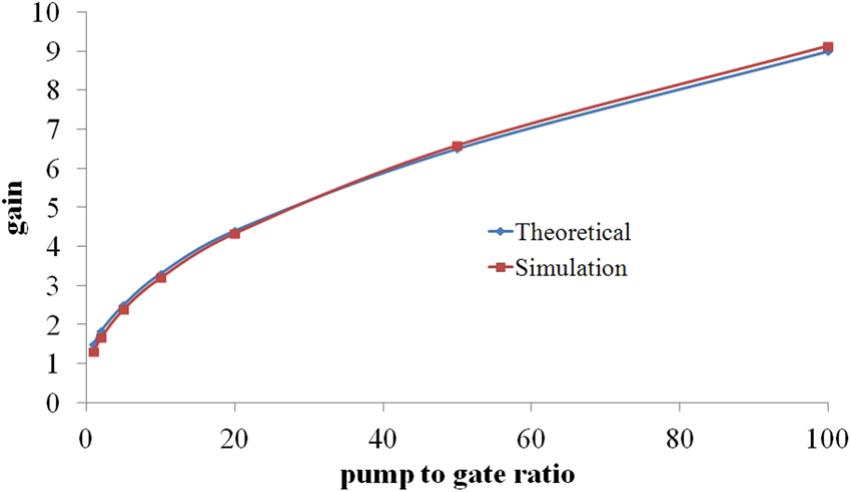
The gain in terms of the pump to gate intensity ratio.

The gain of this system is also organized by the pump to gate intensity ratio as mentioned in Eq. 5. Based on this relation, the gain behavior in terms of the pump to gate intensity ratio should represent a square root behavior. This performance can be deduced from Fig. 7 in which the gain of the transistor with a 25 nm-thick air gap has been depicted against the pump to gate intensity ratio.

**Figure 8:**
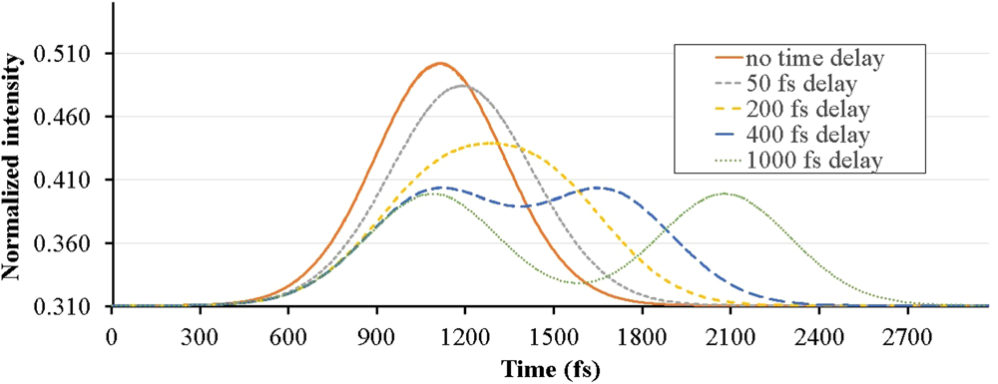
Two consecutive 330 fs pulses overlap in output with different delays.

The time duration in which the optical system returns to the standby mode is another important feature of an optical transistor that determines its switching period. To study this property, we consider the propagation of two similar consecutive pulses. Figure 8 illustrates this propagation for two Gaussian pulses with duration of 330 fs and different delay times. It is obvious that the pulses are distinguishable for a time delay of 400 fs and are completely separated when the delay reaches 1 ps. Therefore the designed PC transistor is capable of operating in terahertz frequencies.

**Figure 9:**
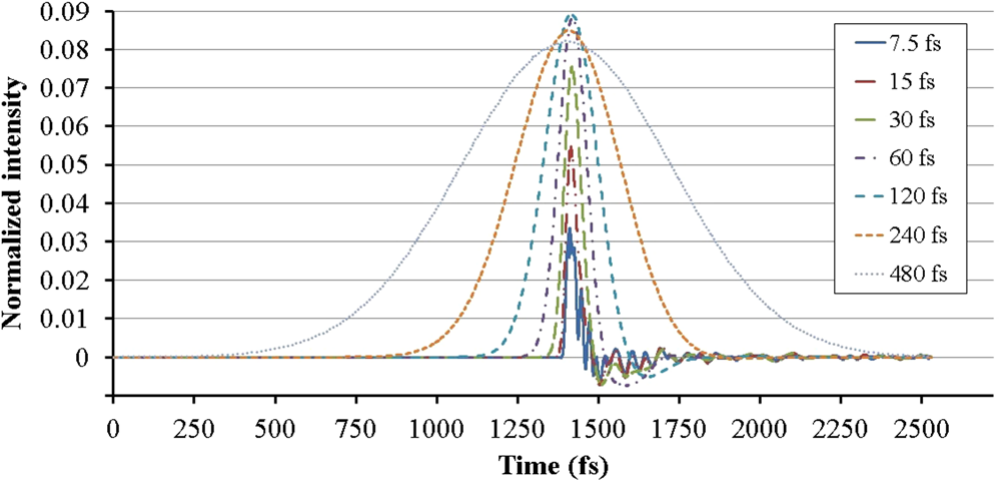
Output pulse for various input pulse lengths.

The quality of the output pulse and the gain of the system are also influenced by the pulse shape. To determine the shortest pulse which can be properly amplified by the optical transistor, we draw the output of the system for gate pulses with different pulse lengths. Figure 9 indicates that to have an acceptable pulse shape at the output, the gate pulse should be longer than 240 fs. For a shorter gate pulse whose length is comparable to the gap size, a fluctuation happens in the tale of the output pulse. This fluctuation is the result of interference of the tunneled and reflected waves.

**Figure 10:**
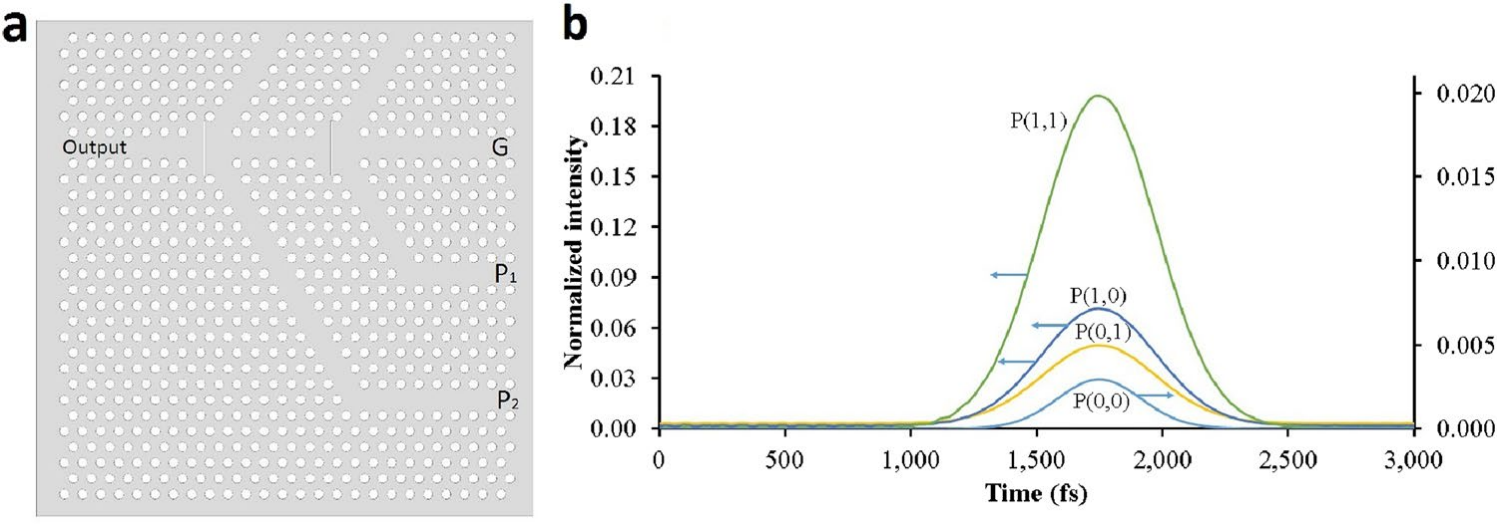
(**a**) The cascade transistor structure and (**b**) its output.

Finally, we study the possibility of making a cascade all-optical transistor from the designed PC structure. In this order, we attach two transistors to each other in a way that the output of the first one will play the role of the input pulse for the second transistor. Concerning the computational system capacity, taking a slab thickness of 700 nm was not possible to evaluate the cascaded transistor performance, and so the simulation was done in two dimensions. Figure 10 illustrates the configuration of this cascade transistor and also its output as an amplified pulse. We can see that this transistor comprises two pump beams, P_1_ and P_2_, and for instance P(1,0) in Fig. 10b, illustrates the situation in which P_1_ is on and P_2_ is off. As mentioned above we could achieve a gain of 9 for a single PC transistor for which we considered the gate to pump intensity ratio of 1/100. To preserve this value of ratio, we fix the pump intensity of the second transistor at 10. According to Fig. 10, the peak value of the pulse in the output of the cascade transistor is about 0.2 that by considering the input pulse amplitude (0.01) the gain of the system will be about 20. This result verifies that the designed PC transistor can be integrated into a serial to make a cascade transistor with a higher gain.

## Conclusions

We have introduced a coherent all-optical transistor based on a photonic crystal structure. The amplification in this structure occurs because of light tunneling through an air gap within the PC structure. Using this method, which is based on the frustrated total internal reflection technique, we could attain an all-optical transistor with an acceptable gain value which can support at least two other transistors in an integrated form. This system operates in terahertz frequency range, exploiting pulses longer than 240 fs.
